# Repeated immune activation with low-dose lipopolysaccharide attenuates the severity of Huntington's disease in R6/2 transgenic mice

**DOI:** 10.1080/19768354.2018.1473291

**Published:** 2018-06-20

**Authors:** Sung Won Lee, Hyun Jung Park, Wooseok Im, Manho Kim, Seokmann Hong

**Affiliations:** a Department of Integrative Bioscience and Biotechnology, Institute of Anticancer Medicine Development, Sejong University, Seoul, Korea; b Department of Neurology, Seoul National University Hospital, Seoul, South Korea

**Keywords:** Huntington’s disease, R6/2 transgenic mice, lipopolysaccharide, Dendritic cells, macrophages

## Abstract

Huntington's disease (HD) is a neurodegenerative disorder caused by a mutation in the huntingtin gene. Previously, therapeutic approaches using anti-inflammatory agents were reportedly not effective for preventing HD progression. Since whether immune responses contribute to the onset of HD is not entirely understood, we herein investigated the role of immune activation in HD using the R6/2 transgenic (Tg) HD model mouse. IL12 production and the expression of costimulatory molecules (e.g. CD86 and CD40) on innate immune cells (DCs and macrophages) were diminished in the disease stage of R6/2 Tg mice. Moreover, the number of adaptive T cells (CD4^+^ and CD8^+^ T cells) and the frequency of effector memory phenotype CD4^+^ T cells were decreased in these mice. These results suggest that the severity of HD is closely related to an impaired immune system and might be reversed by activation of the immune system. Since lipopolysaccharide (LPS), a potent TLR4 agonist, activates immune cells, we evaluated the effect of immune activation on the pathogenesis of HD using LPS. The repeated immune activation with low-dose LPS significantly recovered the impaired immune status back to normal levels and attenuated both severe weight loss and the increased clasping phenotype found in the disease stage of R6/2 Tg mice, consequently resulting in prolonged survival. Taken together, these results strongly indicate that immune activation has beneficial influences on alleviating HD pathology and could provide new therapeutic strategies for HD.

## 1. Introduction

Huntington's disease (HD) is a dominantly inherited neurodegenerative disorder caused by a genetic defect. This condition results from the expansion of cytosine-adenine-guanine (CAG) trinucleotide repeats in exon 1 of the gene encoding huntingtin (HTT) protein, resulting in the expression of mutant HTT (MacDonald et al. 1993). HD patients display typical clinical symptoms, such as uncontrolled movements of the limbs and trunk, cognitive deficits and emotional disturbances (Walker 2007). HTT is expressed in most cells, including neurons, and its mutant form is also shown in the brain, adipose tissue, muscle, and immune system (Lodi et al. 2000; Phan et al. 2009; van der Burg et al. 2009). The R6/2 transgenic (Tg) mouse is the most commonly used HD animal model, which expresses an N-terminal fragment with more than 150 CAG repeats in HTT exon 1 (Mangiarini et al. 1996).

The mammal possesses two types of immune systems that work together to defend the body: (1) innate immunity, which refers to the first line of defense against pathogens, and (2) adaptive immunity, which refers to the antigen-specific protection against a specific pathogen. Dendritic cells (DCs), as innate immune cells, are professional antigen-presenting cells (APCs) that specialize in presenting exogenous and endogenous antigens to CD4^+^ and CD8^+^ T cells, respectively. Activated/matured DCs secrete high levels of a Th1-polarizing cytokines, such as IL12, and express increased levels of costimulatory molecules, including CD86 and CD40, leading to optimal T cell activation and survival (Merad et al. 2013). Although the contribution of DCs to pathogenic or inhibitory effects on auto-inflammatory central nervous system (CNS) disease, such as multiple sclerosis (MS), have been well defined (Zozulya et al. 2010; Lee et al. 2015), little is known about the function of DCs in neurodegenerative diseases, including HD. Macrophages, another type of professional APC, are distributed in various tissues, and particularly the CNS-resident macrophages, called microglia, can participate in the maintenance of neuronal synapses and glutamate uptake in addition to antigen presentation (London et al. 2013).

The pathogenesis of HD has been associated with inflammatory responses elicited by accumulation of mutant HTT proteins due to mitochondrial dysfunction (Bossy-Wetzel et al. 2008; Salminen et al. 2012). The plasma level of pro-inflammatory cytokines, such as IL6 and IL8, were increased in HD patients compared to that in healthy subjects (Bjorkqvist et al. 2008). Treatment of anti-inflammatory minocycline, a second-generation tetracycline (Bastos et al. 2007), delays HD progression in the R6/2 Tg mice (Chen et al. 2000). However, some studies have reported that minocycline cannot protect mice from HD (Smith et al. 2003; Mievis et al. 2007). Moreover, anti-inflammatory treatment with acetylsalicylate or rofecoxib failed to protect the progression of HD (Norflus et al. 2004), suggesting that inflammatory condition might be a sign rather than a direct critical factor causing the disease.

Here, we demonstrated that R6/2 Tg mice with the onset of HD show a noticeably attenuated immune status whereas mice with pre-manifest HD display an immune status comparable to that of wild-type (WT) littermate control mice, regarding both innate and adaptive immune responses. Moreover, the treatment of HD-onset R6/2 Tg mice with low-dose lipopolysaccharide (LPS) stimulated innate immune cells, including DCs, leading to activation of T cells, ultimately delaying disease progression. Therefore, our results provide evidence supporting the preventive effects of low levels of inflammation on HD pathogenesis.

## 2. Materials and methods

### 2.1. Mice

HD Tg mice of the R6/2 line (B6CBA-Tg(HDexon1)62Gpb/3J; hereafter R6/2 Tg mice) were purchased from the Jackson Laboratory. All mice in the present study were maintained at Sejong University and used for experiments at 6–12 weeks of age. The mice were maintained on a 12-hour light/12-hour dark cycle in a temperature-controlled barrier facility with free access to food and water. The mice were fed a γ-irradiated sterile diet and provided autoclaved tap water. Age- and sex-matched R6/2 Tg and WT littermate control mice were used for all experiments. The animal experiments were approved by the Institutional Animal Care and Use Committee at Sejong University (SJ-20160704). The present experiments were conducted in a blinded and randomized trial.

### 2.2. Intraperitoneal injection of LPS into mice for inducing immune activation

LPS derived from *Escherichia coli* (serotype 0111:B4) was purchased from Sigma-Aldrich (St. Louis, MO, USA). For survival experiments, R6/2 Tg mice were intraperitoneally (i.p.) injected with LPS (2 μg) dissolved in phosphate buffered saline (PBS) once a week starting from 5 weeks of age for 12 weeks. Littermate R6/2 Tg mice injected with PBS were only used as negative controls. The survival rate for both groups of mice was monitored and recorded every week after injection. In addition, R6/2 Tg mice injected once a week with either LPS (2 μg) or PBS (control) were sacrificed after a total of 8 injections and subjected to immunological analysis.

### 2.3. Genotyping of mice

To confirm the presence of HTT mutant transgene, genomic DNA from tail biopsies was used to amplify a 170 bp fragment that was only detectable in mice carrying the HD transgene. The following primers were used for PCR genotyping: forward 5′-CCG CTC AGG TTC TGC TTT TA-3′; and reverse 5′-TGG AAG GAC TTG AGG GAC TC-3′.

### 2.4. Mouse brain isolation and clasping test

The mice were anesthetized by using ketamine and xylazine (40 and 4 mg/kg, respectively) and were perfused through the left cardiac ventricle with cold PBS (pH = 7.4) for 3 min to remove cells from the blood vessels. The brain was removed. For testing clasping, 4- or 12-week-old mice were suspended by the tail for 30 s, and the foot-clasping time was scored as follows: 3, >10 s; 2, 5–10 s; 1, 0–5 s; 0, 0 s (Nguyen et al. 2005).

### 2.5. Cell isolation and culture

A single-cell suspension of splenocytes was prepared and resuspended in RPMI complete medium consisting of RPMI 1640 (Gibco BRL, USA) medium supplemented with 10% FBS, 10 mM HEPES, 2 mM L-glutamine, 100 units/mL penicillin-streptomycin, and 5 mM 2-mercaptoethanol.

### 2.6. Flow cytometry

The following monoclonal antibodies (mAbs) from BD Biosciences were used: fluorescein isothiocyanate (FITC)-, phycoerythrin (PE)-Cy7-, or allophycocyanin (APC)-conjugated anti-CD3ϵ (clone 145-2C11); FITC- or PE-Cy7-conjugated anti-CD4 (clone RM4-5); FITC- or APC-conjugated anti-CD11c (clone HL3); PE-Cy7-conjugated anti-CD11b (clone M1/70); PE-conjugated anti-CD62L (clone MEL-14); PE-Cy7-conjugated anti-CD8 (clone 53-6.7); biotin-conjugated anti-CD86 (clone GL1); PE-conjugated anti-IL12p40 (clone C15.6); and FITC- or PE-conjugated anti-IgG1 (isotype control) (clone R3-34). The following mAbs from eBioscience (San Diego, CA, USA) were used: APC-conjugated anti-F4/80 (clone BM8); PE-conjugated anti-CD40 (clone 3/23). To perform surface staining, cells were harvested and washed twice with cold 0.5% BSA-containing PBS (FACS buffer). To block Fc receptors, the cells were incubated with anti-CD16/CD32 mAbs on ice for 10 min and subsequently stained with fluorescence-labeled mAbs. Flow cytometric data were acquired using a FACSCalibur flow cytometer (Becton Dickson, San Jose, CA, USA) and analyzed using FlowJo software (Tree Star Inc., Ashland, OR, USA).

### 2.7. Intracellular cytokine staining

For intracellular staining, splenocytes were incubated with brefeldin A, an intracellular protein transport inhibitor (10 μg/ml), in RPMI medium for 2 h at 37°C. The cells were stained for cell surface markers, fixed with 1% paraformaldehyde, washed once with cold FACS buffer, and permeabilized with 0.5% saponin. The permeabilized cells were then stained for an additional 30 min at room temperature with the indicated mAbs (PE-conjugated anti-IL12, or PE-conjugated isotype control rat IgG mAbs). More than 5000 cells per sample were acquired using a FACSCalibur, and the data were analyzed using the FlowJo software package (Tree Star, Ashland, OR, USA).

### 2.8. Statistical analysis

Statistical significance was determined using Excel (Microsoft, USA). Student's *t*-test was performed for the comparison of two groups. *P < 0.05, **P < 0.01, and ***P < 0.001 were considered to be significant in the Student's *t*-test. Two-way ANOVA analysis was carried out using the VassarStats (http://faculty.vassar.edu/lowry/VassarStats.html). ^#^*P* < 0.05, ^##^*P* < 0.01, and ^###^*P* < 0.001 were considered to be significant in the two-way ANOVA.

## 3. Results

### 3.1. Dramatic reduction in splenic immune cells at the disease stage of R6/2 Tg mice

**Figure 1.**
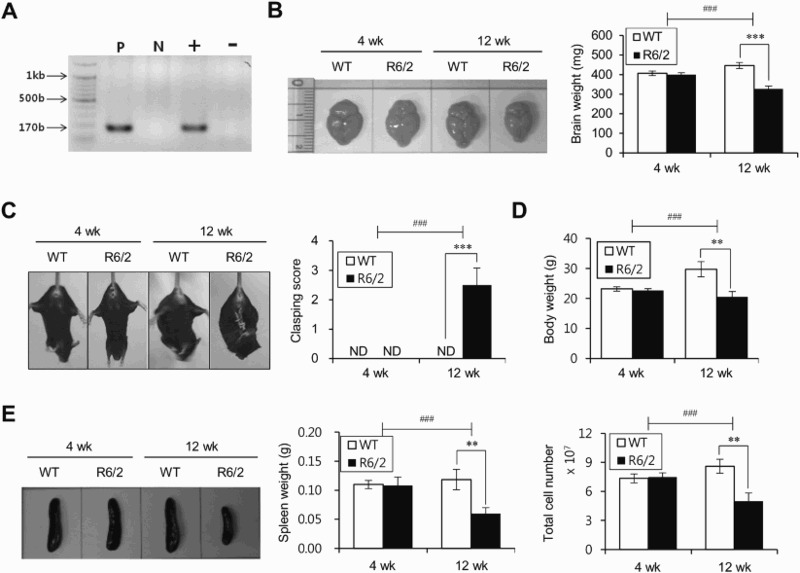
HD-onset R6/2 Tg mice show reduced levels of splenocytes. (A) Genotyping HD mutant gene by PCR analysis. HTT mutant PCR band sizes 170 bp long. M: marker; Lane 1: positive control; Lane 2: negative control; Lane 3: HD positive; Lane 4: HD negative. (B) Brains were harvested from WT littermates or R6/2 Tg mice of 4 or 12 weeks of age (left panel) and their brain weights were evaluated (right panel). (C) Clasping test and (D) body weight were evaluated from WT littermates and R6/2 Tg mice of 4 or 12 weeks of age. (E) Spleens were prepared from WT littermates or R6/2 Tg mice of 4 or 12 weeks of age and the spleen weight and splenocyte number were evaluated. The mean values ± SD (*n* = 4 in B, C, D, and E; per group in the experiment; Student's *t*-test; **P < 0.01, ***P < 0.001) are shown. Two-way ANOVA (genotype × time) showed an interaction between these two factors (^#^*P* < 0.05, ^##^*P* < 0.01, and ^###^*P* < 0.001).

The R6/2 Tg mouse is a well-characterized animal model for HD, which represents behavioral, neuronal, and pathological properties of the disease (Mangiarini et al. 1996). To confirm the characteristics during natural disease progression in R6/2 Tg mice, we compared the disease phenotypes of R6/2 Tg mice with those of the WT littermate control mice at either 4 or 12 weeks of age. The brains of 12-week-old but not 4-week-old R6/2 Tg mice weighed approximately 25% less than those from WT littermates of the same age (Figure 1B), indicating that 12-week-old R6/2 Tg mice showed a more progressed disease phenotype. Next, we compared the clasping behavior and body weight between R6/2 Tg mice and WT littermates at either 4 or 12 weeks of age. Twelve-week-old but not 4-week-old R6/2 Tg mice exhibited a substantial increase of clasping phenotype (Figure 1C) and displayed decreased body weight compared with WT littermates of the same age (Figure 1D).

Since changes in immune responses have been correlated with the severity of HD (Soulet & Cicchetti 2011), we compared the spleen weight as well as the total number of splenocytes during HD development in R6/2 Tg mice. Unlike 4-week-old R6/2 Tg mice, both the spleen weight and the total number of splenocytes were significantly decreased in 12-week-old R6/2 Tg mice compared to those of their WT littermates (Figure 1E). These data indicated that the progression of HD into late-stage is strongly associated with reduced immune activation.

### 3.2. HD-onset R6/2 Tg mice exhibit impaired functions of APCs, consistent with reduced levels of T cell immune responses

**Figure 2.**
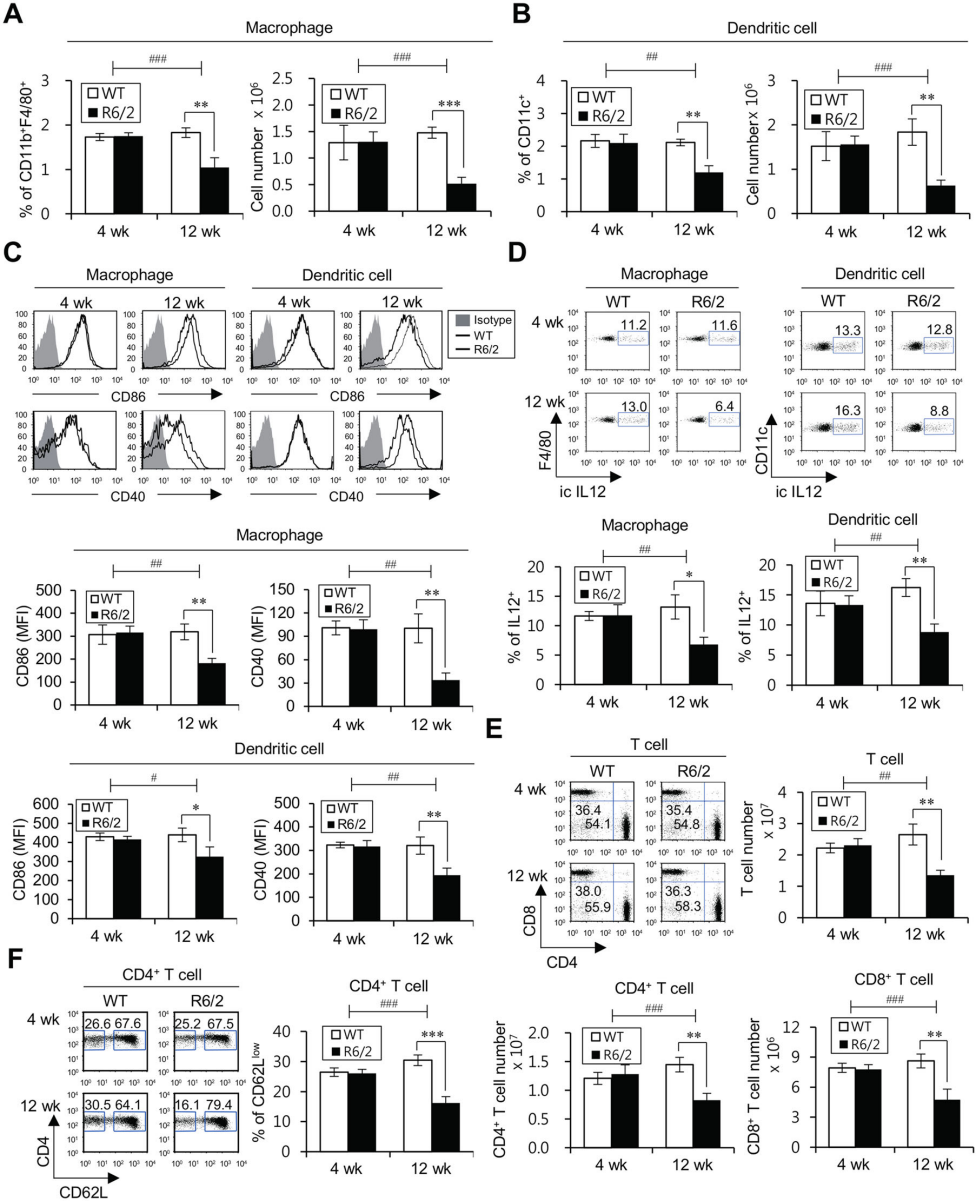
HD-onset R6/2 Tg mice exhibit impaired functions of APCs, concordant with reduced levels of T cell immune responses. (A-D) Splenocytes were isolated from the spleens of WT littermates and R6/2 Tg mice at either 4 or 12 weeks of age. (A) The percentage of CD11b^+^F4/80^+^ macrophages among CD11c^-^ splenic populations was evaluated via flow cytometry (left panels). The absolute cell number of macrophages was determined (right panels). (B) The percentage of CD11c^+^ splenic population was evaluated via flow cytometry (left panels). The absolute cell number of DCs was determined (right panels). (C) Surface expressions of CD86 and CD40 and (D) intracellular IL12 production were analyzed in macrophages (CD11b^+^F4/80^+^CD11c^-^) and DCs (CD11c^+^). *Top*, representative FACS plot; *bottom*, summary figures. The mean values ± SD (*n* = 4 in A, B, C, and D; per group in the experiment; Student's *t*-test; *P < 0.05, **P < 0.01, ***P < 0.001) are shown. Two-way ANOVA (genotype × time) showed an interaction between these two factors (^#^*P* < 0.05, ^##^*P* < 0.01, and ^###^*P* < 0.001). (E-F) Splenocytes were isolated from the spleens of WT littermates and R6/2 Tg mice at either 4 or 12 weeks of age. (E) The frequencies of total T cells, CD4^+^ T cells, and CD8^+^ T cells were calculated by gating on CD3^+^, CD3^+^CD4^+^CD8^-^, and CD3^+^CD4^-^CD8^+^ populations, as observed in the upper left panels. (F) The frequencies of effector/memory CD4^+^ T cells and naive CD4^+^ T cells were calculated by gating on CD3^+^CD4^+^CD62L^low^ and CD3^+^CD4^+^CD62L^high^ populations, as observed in the left panels. The mean values ± SD (*n* = 4 in E and F; per group in the experiment; Student's *t*-test; **P < 0.01, ***P < 0.001) are shown. Two-way ANOVA (genotype × time) showed an interaction between these two factors (^##^*P* < 0.01, and ^###^*P* < 0.001).

As DCs and macrophages are involved in the regulation of CNS function other than the induction of T cell polarization (Zozulya et al. 2010; London et al. 2013; Merad et al. 2013), we next examined whether the progressed HD phenotype is closely related with alteration of APC number and function. The frequency and absolute number of these cells were significantly reduced in 12-week-old but not 4-week-old R6/2 Tg mice (Figure 2A and B). Next, to determine whether there is any attenuation of DC activation, we measured the expression level of activation markers, including CD86, CD40, and IL12, in R6/2 Tg mice. Unlike no difference in the expression of costimulatory molecules, CD86 and CD40, between R6/2 Tg mice and WT littermates at pre-manifest stage HD (4-week-old), the expression levels of these molecules were significantly decreased in R6/2 Tg mice at late stage HD (12-week-old) compared to that in WT littermates (Figure 2C). Additionally, IL12 production was diminished in R6/2 Tg mice at 12 weeks of age, suggesting that attenuated immune responses are correlated with the progression of HD (Figure 2D). Our results strongly indicated that both quantitative and qualitative reductions of APCs in HD-onset R6/2 Tg mice were dependent on the stage of disease progression.

Since adaptive immune responses do not occur without innate immune responses, we further investigated whether adaptive immune cells, such as CD4^+^ and CD8^+^ T cells, could be altered by HD progression. In 12-week-old but not 4-week-old R6/2 Tg mice, the number of these cells was significantly decreased compared with that in WT littermates (Figure 2E). Next, to investigate the functionality (activation status) of T cells, we compared the CD62L surface expression (a marker for effector/memory cells) between R6/2 Tg and WT littermate control mice. The frequency of CD62L^low^ effector/memory CD4^+^ T cells was diminished in HD-onset R6/2 Tg mice (Figure 2F). Thus, we provide the evidence that the reduced frequency of effector T cells is correlated with APC defects affected by HD progression.

### 3.3. Low-dose LPS treatment can delay HD progression in R6/2 Tg mice

**Figure 3.**
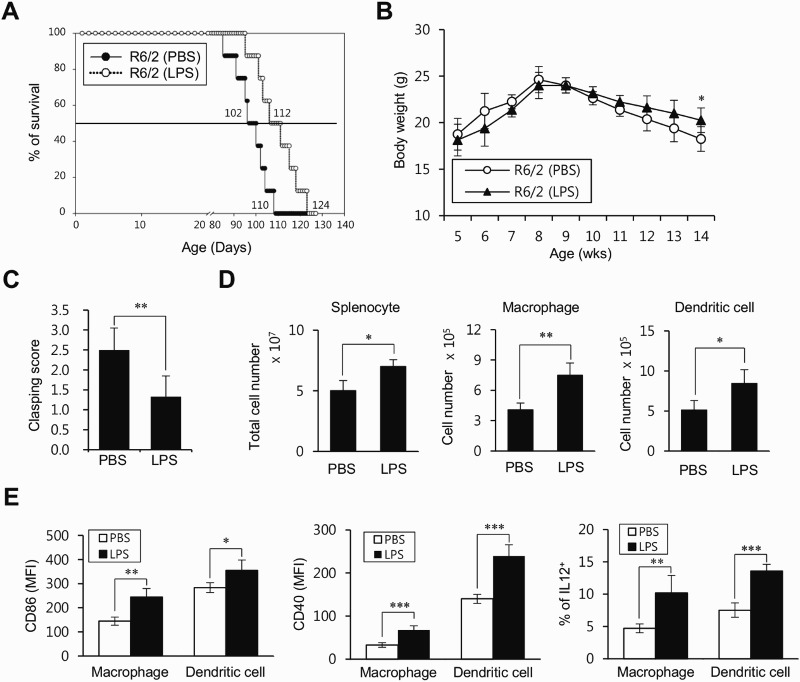
Low-dose LPS treatment prevents HD progression in R6/2 Tg mice. (A) R6/2 Tg mice were i.p. injected with either PBS (*n* = 8) or low-dose LPS (2 μg) (*n* = 8) once per week starting from 5 weeks of age for total 12 weeks. The survival rate of these mice was monitored every week after LPS treatment. (B-E) R6/2 Tg mice were i.p. injected with either PBS (*n* = 4) or low-dose LPS (2 μg) (*n* = 4) treatment from 5 to 14 weeks old. (B) The mice were also weighed weekly from 5 to 14 weeks old. (C) Mice were tested for hind limb clasping behavior at 7 weeks after LPS injection. (D) The absolute cell number of total splenocytes, macrophages, and DCs were determined at 9 weeks after LPS injection. (E) Surface expressions of CD86 and CD40 and intracellular IL12 production were analyzed in macrophages (CD11b^+^F4/80^+^CD11c^-^) and DCs (CD11c^+^) at 9 weeks after LPS injection. The mean values ± SD (*n* = 8 in A; *n* = 4 in B, C, D, and E; per group in the experiment; Student's *t*-test; *P < 0.05, **P < 0.01, ***P < 0.001) are shown.

LPS, which is one of the most potent TLR4 agonists, triggers the activation of innate immune cells (most notably DCs and macrophages) (Mogensen 2009). Thus, to assess the effect of LPS on the course of HD, R6/2 Tg mice were treated with low-dose LPS and were subsequently monitored for phenotypes, including survival, clasping score, and body weight. We found that the survival duration was significantly prolonged in LPS-treated R6/2 Tg mice compared to that in the untreated WT littermate control group (Figure 3A). Body weight was also assessed in R6/2 Tg mice treated with either LPS or vehicle for 9 weeks starting at 5 weeks of age. The weight loss was much less severe in R6/2 Tg mice treated with LPS compared to that in the WT littermate control mice (Figure 3B). Furthermore, the clinical score of clasping phenotype was attenuated in LPS-treated R6/2 Tg mice compared with that in the PBS-treated WT littermate control group (Figure 3C).

Next, we examined whether LPS treatment has any influence on the alteration of innate immune cells in R6/2 Tg mice. The number of splenic macrophages and DCs, as well as total splenocytes, was significantly increased in LPS-treated R6/2 Tg mice compared with those in the PBS-treated WT littermate control group (Figure 3D). Moreover, the expression of costimulatory molecules (CD86 and CD40) and IL12 production were both up-regulated in LPS-treated R6/2 Tg mice compared to that in the PBS-treated WT littermate control group (Figure 3E). Taken together, these results demonstrated that LPS treatment could prevent the pathogenesis of HD through the activation of innate immune responses.

## 4. Discussion

Although previous studies have suggested that pro-inflammatory conditions by immune cells cause an acceleration of HD, the efficacy of anti-inflammatory agents in treating HD has not been satisfied (Norflus et al. 2004). The role of immune activation in HD has not been fully elucidated. Therefore, we aimed to determine whether immune activation modulates the pathogenesis of HD. In the late HD stage of R6/2 Tg mice, the cell number and activation status of adaptive immune cells, as well as innate immune cells (DCs and macrophages), were diminished, suggesting that the severity of HD might be associated with an impaired immune system. Moreover, we showed that immune activation through repeated LPS treatment significantly delayed the progression of HD.

Since glutamate uptake was reduced in the prefrontal cortex of HD patients, it has been proposed that glutamate uptake might be inversely correlated with CAG repeat expansion (Hassel et al. 2008). An increase of glutamate uptake induced by up-regulation of the glutamate transporter 1 (GLT-1) attenuates HD signs in R6/2 Tg mice (Miller et al. 2008). Based on a previous study, showing that LPS treatment increases GLT-1 expression and glutamate uptake in microglia in a TNFα-dependent manner (Persson et al. 2005), the finding that LPS treatment improves the clinical symptoms of HD could be associated with a change in glutamate uptake. Since IFNγ signaling to astrocytes with glutamate clearance is critical for neuroprotection (Hindinger et al. 2012), it is reasonable to speculate that IFNγ derived from LPS-activated immune cells might induce paracrine cytokine stimulation of astrocytes, thereby attenuating the progression of HD. Thus, it will be of interest to investigate this issue in the future.

N171-82Q HD mice were recently reported to have fewer IFNγ-producing CD8^+^ T cells in response to *Toxoplasma gondii* infection, leading to premature death, suggesting that the immune system in HD mice is downregulated (Donley et al. 2016). Consistently, the present results showed that the late-symptomatic R6/2 Tg mice displayed significantly reduced levels of inflammatory responses. Additionally, the majority of HD patients failed to control pneumonia infection, which becomes, in fact, the most common cause of death (Heemskerk & Roos 2012). Thus, the adjuvant effects of LPS on the immune system could be useful to delay or prevent progression to advanced HD stage in patients.

Although mutant HTT is expressed in the brain, which is the primary pathological tissue of HD, mutant HTT is expressed in other tissues (e.g. fat and muscle) and immune system (Phan et al. 2009; van der Burg et al. 2009; Soulet & Cicchetti 2011). It has previously been reported that mutant HTT was expressed in immune cells and their migratory functions were consequently impaired in peripheral myeloid cells, such as monocytes, as well as microglia from pre-manifest human HD patients, suggesting that immune dysfunction may be attributed to poly-glutamine expansion (Kwan et al. 2012). Moreover, a recent study provided clear evidence that the immune system can restrain the pathogenesis of Alzheimer's disease, another type of neurodegenerative disease, via the modulation of microglial function (Marsh et al. 2016). Our results suggest that downregulated immune functions are closely correlated with the full-blown disease status of HD and indicate that the adoptive transfer of functional immune cells (with normal HTT gene) into HD patients might be beneficial by retarding disease progression.

In conclusion, our study provides *in vivo* evidence that the repeated low-dose LPS treatment could awake the inhibited (or anergized) peripheral immune system, which delays or prevents the further progression of HD. These findings will help to design a new therapeutic strategy to modulate HD pathology.
